# Supplementary material for: Are the Items of the Starkstein Apathy Scale Fit for the Purpose of Measuring Apathy Post-stroke?
Source: Front Psychol. 2021 Dec 7;12:754103. doi: 10.3389/fpsyg.2021.754103 (PMC8688540; doi:10.3389/fpsyg.2021.754103)
Supplement: Supplementary file 1 [file Data_Sheet_1.pdf]

Appendix

Figure 1a. Threshold distances varied across items

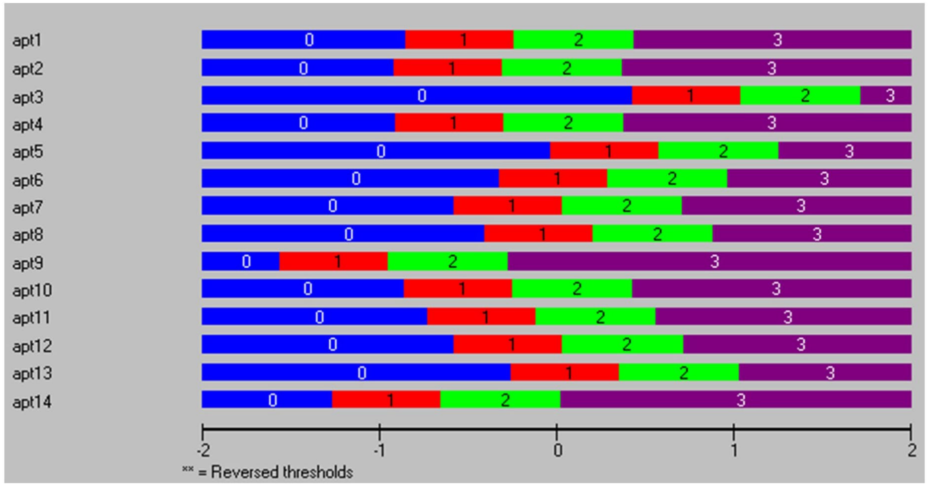

Table Ia. Summary fit statistics for Starkstein's Apathy Scale
based on 5 random sample each with n=300 and baseline time point

Model	sample size (n)	Overall model fit	Item Location	Item fit residual Mean (SD)	Person Location	Person fit residual Mean (SD)	PSI	Unidimensionality *t*-tests %[a] (95%CI)
sample1	282	X^2=39.547, df=45, p=0.701	0.0 (0.457)	-0.137 (1.095)	1.680 (1.279)	-0.293 (1.095)	0.74	4.96 (2.98-8.16)
sample2	283	X^2=56.644, df=45, p=0.114287	0.0 (0.495)	-0.148 (1.010)	1.656 (1.298)	-0.356 (1.190)	0.74	4.59 (2.70-7.70)
sample3	284	X^2=58.835, df=45, p=0.080793	0.0 (0.453)	-0.122 (0.976)	1.612 (1.225)	-0.315 (1.132)	0.72	3.87 (2.18-6.80)
sample4	287	X^2=47.047, df=45, p=0.388697	0.0 (0.412)	-0.172 (0.978)	1.479 (1.316)	0.303 (1.183)	0.75	6.62 (4.28-10.11)
sample5	282	X^2=56.119, df=45, p=0.123756	0.0 (0.443)	0.033 (1.149)	1.562 (1.198)	-0.328 (1.193)	0.72	2.84 (1.44-5.50)
baseline 9 items	225	X^2=31.004, df=27, p=0.270978	0.0 (0.449)	-0.001 (0.938)	1.372 (1.219)	-0.341 (1.349)	0.75	5.33 (3.08-9.09)

The average p-value for the 5 random samples was 0.28 with an average PSI=0.73.

SD standard deviation, PSI person separation index, df degrees of freedom

[a]% of statistically significant *t*-tests

Table 1b. Summary of DIF results for the 5 random samples and the baseline time point

DIF	sample 1	sample 2	sample 3	sample 4	sample 5	Baseline time point
time	no	no	no	no	item #4 p=0.001080	no
age	no	no	no	no	item #4 p=0.000900	no
sex	item #4 p=0.001116	item #8 p=0.000705; item #10 p=0.000956	item #9 p=0.000373	item #8 p=000662; item #9 p=0.000117	item #1 p=0.000758	no
edu2	no	no	item #1 p=0.001838	no	no	no
edu4	no	no	no	no	no	no

Bonferroni probability adjustment at the 0.002778 signifance level

Table IIa. Loading on first component of the principal component analysis of the item residuals of the SAS

all (14) items	Item description	PC1: Loading on first component extracted	9 item model	Item description	PC1: Loading on first component extracted	4 items (#3, #13, #5, #5)	Item description	PC1: Loading on first component extracted
I0011	Are you unconcerned with many things?	0.534	I0001	Are you interested in learning new things?	0.55	I0003	Are you concerned about your condition?	0.799
I0010	Are you indifferent to things?	0.525	I0002	Does anything interest you?	0.47	I0013	Are you neither happy nor sad, just in between?	0.425
I0013	Are you neither happy nor sad, just in between?	0.42	I0004	Do you put much effort into things?	0.332	I0006	Do you have plans and goals for the future?	-0.644
I0009	Does someone have to tell you what to do each day?	0.412	I0007	Do you have motivation?	0.308	I0005	Do you have plans and goals for the future?	-0.699
I0003	Are you concerned about your condition?	0.312	I0008	Do you have the energy for daily activities?	0.178			
I0014	Would you consider yourself apathetic?	0.287	I0014	Would you consider yourself apathetic?	-0.397			
I0012	Do you need a push to get started on things?	0.242	I0009	Does someone have to tell you what to do each day?	-0.473			
I0002	Does anything interest you?	-0.217	I0012	Do you need a push to get started on things?	-0.512			
I0008	Do you have the energy for daily activities?	-0.275	I0010	Are you indifferent to things?	-0.517			
I0001	Are you interested in learning new things?	-0.365						
I0004	Do you put much effort into things?	-0.386						
I0006	Do you have plans and goals for the future?	-0.435						
I0005	Do you have plans and goals for the future?	-0.44						
I0007	Do you have motivation?	-0.464						

Table IIb. Summary statistics for 4 items (misfitting items deleted from the SAS)

Model	sample size (n)	Overall model fit	Item Location	Item fit residual Mean (SD)	Person Location	Person fit residual Mean (SD)	PSI	item fit	[a]% total variance by first component
4 items (#3, #13, #6, #5)	842	X^2=68.439, df=12, p=0.000000	0.0 (0.388)	1.610 (1.807)	0.221 (0.805)	-0.410 (1.450)	0.06	item #3 fit residual 4.3	43.07
3 items (deleted item 3)	784	X^2=40.251, df=9, p=0.000007	0.0 (0.231)	1.691 (1.443)	0.405 (1.021)	-0.669 (1.704)	0.22	item #13 fit residual 3.4	53.58

SD standard deviation, PSI person separation index, df degrees of freedom

[a]% of total variance explained by the first component of the Principal Component Analysis of the Residuals after the Rasch factor has been extracted.
